# Supplementary material for: Chemical Structures of Lignans and Neolignans Isolated from Lauraceae
Source: Molecules. 2018 Nov 30;23(12):3164. doi: 10.3390/molecules23123164 (PMC6321345; doi:10.3390/molecules23123164)
Supplement: Supplementary file 1 [file molecules-23-03164-s001.pdf]

# Chemical Structures of Lignans and Neolignans Isolated from Lauraceae

**Ya Li [1,*], Shuhan Xie [2], Jinchuan Ying [1], Wenjun Wei [1] and Kun Gao [1,*]**

[1] State Key Laboratory of Applied Organic Chemistry, College of Chemistry and Chemical Engineering, Lanzhou University, Lanzhou 730000, P. R. China; liea@lzu.edu.cn (Y. L.); yingjch16@lzu.edu.cn (J.C. Y.); weiwj14@lzu.edu.cn (W.J. W); npchem@lzu.edu.cn (K. G.)
[2] Lanzhou University High School, Lanzhou 730000, P. R. China; xieshzb@qq.com
* Correspondences: liea@lzu.edu.cn (Y. L.); npchem@lzu.edu.cn (K. G.).

**Table SI-1.** Semi-systematic names and names in references of lignans and cyclolignans

| No. | Semi-systematic names | Names in references | Ref. |
|---|---|---|---|
| **5** | *meso*-3,4,5,3',4',5'-hexamethoxylignan | *meso*-3,4,5,3',4',5'–hexamethoxy-8.8'-lignan | [6] |
| **6** | *threo*-3,4,5,3',4',5'-hexamethoxylignan | *threo*-3,4,5,3',4',5'–hexamethoxy-8.8'-lignan | [6] |
| **12** | *threo*-4,9,4',9'-Tetrahydroxy-3,5,3',5'-tetramethoxylignan | 2,3-bis[(4-hydroxy-3,5-dimethoxyphenyl)-methyl]-1,4-butanediol | [10] |
| **13** | (7*R*,8*S*,8'*R*)-7-hydroxy-3,4,3',4'- tetramethoxylignan | (7*R*,8*S*,8'*R*)-7-hydroxy-3,4,3',4'-tetramethoxy-8.8'-neolignan | [11] |
| **14** | (8*S*,8'*R*)-4'-hydroxy-3'-methoxy-3,4-methylene dioxylign-7-one | (8*S*,8'*R*)-4'-hydroxy-3'-methoxy-3,4-methylenedioxy-7-oxo-8.8'-neolignan | [11] |
| **15** | (8*S*,8'*R*)-3.4,3'.4'-dimethylenedioxy-lign-7-one | (8*S*,8'*R*)-3.4,3'.4'-dimethylenedioxy-7-oxo-8.8'-neolignan | [11] |
| **16** | (8*S*,8'*R*)-3',4'-dimethoxy-3,4-methylenedioxylign-7-one | (8*S*,8'*R*)-3',4'-dimethoxy-3,4-methylenedioxy-7-oxo-8.8'-neolignan | [11] |
| **27** | *rel*-(7*S*,8*S*,7'*S*,8'*R*)-7,7'-epoxy-2,5,3',4',5'-pentamethoxylignane | 2-(2',5'-dimethoxyphenyl)-3,4-dimethyl-5-(3", 4", 5"-trimethoxyphenyl)-tetrahydrofuran | [14] |
| **28** | *rel*-(7*S*,8*S*,7'*S*,8'*R*)-7,7'-epoxy-2,5,3',4'-tetramethoxylignane | 2-(2',5'-dimethoxyphenyl)-3,4-dimethyl-5-(3", 4", 5"-trimethoxyphenyl)-tetrahydrofuran | [14] |
| **43** | (7*R*,8*R*,7*R*',8'*R*)-2.9,2'.9'-diepoxy-7,7'-dihydroxy-3,3'-dimethoxylignan | (3*R*,4*R*,3'*R*,4*R*')-6,6'-dimethoxy-3,4,3',4'-tetrahydro-2H,2'H[3, 3']bichromenyl-4, 4'-diol | [24] |
| **65** | (8*R*)-3,4-dimethoxy-3',4'-methylenedioxy-2.7',8.8'-cyclolign-7'-ene | (8R)-3,4-dimethoxy-3',4'-methylenedioxy-$\Delta^{7',8'}$-6.7',8.8'-neolignan | [28] |
| **66** | 4-hydroxy-3-methoxy-3',4'-methylenedioxy-2.7',8.8'-cyclolign-7,7'-diene | 4-hydroxy-3-methoxy-3',4'-methylenedioxy-$\Delta^{7,8,7',8'}$-6.7',8.8'-neolignan | [28] |

**Table SI-2.** Semi-systematic names and names in references of 8,1'-neolignans

| No. | Semi-systematic name | Name in reference | Ref. |
|---|---|---|---|
| **72** | (7*S*,8*S*,1'*R*)-7,2'-epoxy-3,4,5'-trimethoxy-8,1'-neolign-8'-ene-4'(1'H)-one | (2*S*,3*S*,3a*R*)-3a-allyl-5-methoxy-3-methyl-2-veratryl-2,3,3a,6-tetrahydro-6-oxobenzofuran | [40] |
| **73** | (7*S*,8*S*,1'*R*)-7,2'-epoxy-3,4,3',5'-tetramethoxy-8,1'-neolign-8'-ene-4'(1'H)-one | (2*S*,3*S*,3a*R*)-3a-allyl-5,7-dimethoxy-3-methyl-2-veratryl-2,3,3a,6-tetrahydro-6-oxobenzofuran | [40] |
| **74** | (7*S*,8*S*,1'*R*)-7,2'-Epoxy-3,4,5,5'-tetramethoxy-8,1'-neolign-8'-ene-4'(1'H)-one | (7S,8S,l′*R*)-$\Delta^{8'}$-3,4,5,5′-tetramethoxy-1′,4′-dihydro-4′-oxo-7.O.2′,8.l′-neolignan | [41] |
| **76** | (7*R*,8*S*,1'*S*)-7,2'-epoxy-5'-methoxy-3,4-methylenedioxy-8,1'-neolign-8'-ene-4'(1'H)-one | (2*R*,3*S*,3a*S*)-3a-allyl-5-methoxy-3-methyl-2-piperonyl-2,3,3a,6-tetrahydro-6-oxobenzofuran | [42, 43] |
| **77** | (7*R*,8*S*,1'*S*)-7,2'-epoxy-3',5'-dimethoxy-3,4-methylenedioxy-8,1'-neolign-8'-ene- 4'(1'H)-one | (7*R*,8*S*,1'*S*)-$\Delta^{8'}$-3',5'-dimethoxy-3,4-methylenedioxy-1′,4′-dihydro-4′-oxo-7.O.2′,8.l′-neolignan | [44, 45] |
| **78** | *rel*-(7*R*,8*S*,1'*S*)-7,2'-epoxy-3,5'-dimethoxy-4,5-methylenedioxy-8,1'-neolign-8'-ene-4'(1'H)-one | *rel*-(2*R*,3*S*,3a*S*)-3a-allyl-5-methoxy-2-(3-methoxy-4,5-methylenedioxyphenyl)-3-methyl-2,3,3a,6-tetrahydro -6-oxobenzofuran | [46, 47] |
| **79** | *rel*-(7*R*,8*S*,1'*S*)-7,2'-epoxy-3,4,5,5'-tetramethoxy-8,1'-neolign-8'-ene-4'(1'H)-one | *rel*-(2*R*,3*S*,3a*S*)-3a-allyl-5-methoxy-2-(3,4,5-trimethoxyphenyl)-3-methyl-2,3,3a,6-tetrahydro-6-oxobenzofuran | [46, 47] |
| **80** | *rel*-(7*S*,8*S*,1'*S*)-7,2'-epoxy-3,4,5,5'-tetramethoxy-8,1'-neolign-8'-ene-4'(1'H)-one | *rel*-(2*S*,3*S*,3a*S*)-3a-allyl-5-methoxy-2-(3,4,5-trimethoxyphenyl)-3-methyl-2,3,3a,6-tetrahydro-6-oxobenzofuran | [46, 47] |
| **85** | (7*S*,8*R*,1'*S*,5'*S*)-7,2'-epoxy-5',6'-dihydro-3,3',5'-trimethoxy-4,5-methylenedioxy-8,1'-neolign-8'-ene - 4'(1'H)-one | (7*S*,8*R*,1′*S*,5′*S*)-$\Delta^{8'}$-3,3′,5′-trimethoxy-4,5-methylenedioxy -1′,4′,5′, 6′-tetrahydro-4′-oxo-7.O.2′,8.l′-neolignan | [50] |
| **89** | *rel*-(7*R*,8*R*,1'*R*,5'*R*)-7,2'-epoxy-5',6'-dihydro-5'-methoxy-3,4-methylenedioxy-8,1'-neolign-8'-ene -4'(1'H)-one | rel-(7*R*,8*R*,1'*R*,5'*R*)-$\Delta^{8'}$-5'-methoxy-3,4-methylenedioxy 1′,4′,5′,6′,-tetrohyhydro-4′-oxo-7.O.2′,8.1′-neolignan | [57, 58] |
| **92** | (7*R*,8*S*,1'*R*,5'*R*)-7,2'-epoxy-5',6'-dihydro-3,4,3',5'-tetramethoxy-8,1'-neolign-8'- ene-4'(1'H)-one | (7*R*,8*S*,l′*R*,3′*R*)-$\Delta^{5',8'}$-3,4,3′,5′-tetramethoxy-4′-oxo-8.1′,7.O.6′-neolignan | [45] |
| **93** | (7*R*,8*S*,1'*R*,5'*R*)-7,2'-epoxy-5',6'-dihydro-3',5'-dimethoxy-3,4-methylenedioxy-8,1'-neolign-8'-ene-4'(1'H)-one | (7*R*,8*S*,l′*R*,3′*R*)-$\Delta^{5',8'}$-3′,5′-dimethoxy-3,4-methylenedioxy-4′-oxo-8.1′,7.O.6′-neolignan | [45] |
| **94** | *rel*-(7*R*,8*S*,1'*R*,5'*S*)-7,2'-epoxy-5',6'-dihydro-5'-methoxy-3,4-methylenedioxy-8,1'-neolign-8'-ene -4'(1'H)-one | *rel*-(7*R*,8*S*,1'*R*,5'*S*)-$\Delta^{8'}$-5′-methoxy-3,4-methylenedioxy -1′,4′,5′,6′-tetrahydro-4′-oxo-7.O.2′,8.l′-neoiignan | [57] |
| **103** | *rel*-(7*S*,8*S*,1'*S*,2'*S*)-7,2'-epoxy-2',3'-dihydoxy-1',2', 3',6'-tetrahydro-5'-methoxy-3,4-methylenedioxy-8,1'- neolign-8'-ene | *rel*-(7*S*,8*S*,1'*S*,2'*S*))-$\Delta^{8'}$-2′,3′-dihydroxy-3,4-methylenedioxy-l′,2′,3′,6′-tetrahydro-7.O.2′,8.1′-neolignan | [57] |
| **104** | *rel*-(7*S*,8*S*,1'*S*,2'*S*)-7,2'-epoxy-3'-hydoxy-1',2',3',6'-tetrahydro-2'-methoxy-3,4-methylenedioxy-8,1'-neolign-8'-ene | *rel*-(7*S*,8*S*,1'*S*,2'*S*))-$\Delta^{8'}$-3′-hydroxy-2′-methoxy-3,4-methylenedioxy-l′,2′,3′,6′-tetrahydro-7.O.2′,8.1′-neolignan | [57] |
| **105** | *rel*-(7*R*,8*S*,1'*R*,2'*S*,4'*R*,5'*R*)-7,2'-epoxy-2',4'-dihydoxy-1',2',3',4',5',6'-hexahydro-3,4,5'-trimethoxy-8,1'- neolign-8'-ene | *rel*-(7*R*,8*S*,1'*R*,2'*S*,4'*R*,5'*R*)-$\Delta^{8'}$-2′,4′-dihydroxy-3,4,5'-trimethoxy-1′,2′,3′,4′,5′,6′-hexahydro-7.O.2′,8.1′-neolignan | [57] |

| | | | |
|---|---|---|---|
| **106** | *rel*-(7*R*,8*S*,1'*R*,2'*S*,4'*S*,5'*R*)-7,2'-epoxy-2',4'-dihydox-1',2',3',4',5',6'-hexahydro-3,4,5'-trimethoxy-8,1'- neolign-8'-ene | *rel*-(7*R*,8*S*,1'*R*,2'*S*,4'*S*,5'*R*)-$\Delta^{8'}$-2′,4′-dihydroxy-3,4,5'-trimethoxy-1′,2′,3′,4′,5′,6′-hexahydro-7.O.2′,8.1′-neolignan | [57] |
| **107** | *rel*-(7*R*,8*S*,1'*R*,2'*S*,4'*R*,5'*R*)-7,2'-epoxy-4'-hydoxy-1',2',3',4',5',6'-hexahydro-3,4,2',5'-tetramethoxy-8,1'-neolign-8'-ene | *rel*-(7*R*,8*S*,1′*R*,3′*R*,4′*R*,6′*S*)-$\Delta^{8'}$-4′-hydroxy-3,4,3′,6′-tetramethoxy-8.1′,7.O.6′-neolignan | [59] |
| **108** | *rel*-(7*R*,8*S*,1'*R*,2'*R*,3'*S*,4'*R*,5'*R*)-7,2'-epoxy-2',3',4'-trihydoxy-1',2',3',4',5',6'-hexahydro-3,4,5'-trimethoxy-8,1'-neolign-8'-ene (**108**), | *rel*-(7*R*,8*S*,1′*R*,3′*R*,4′*R*,5′*S*,6′*R*)-$\Delta^{8'}$-4′,5′,6′-trihydroxy-3,4,3′-trimethoxy-8.1′,7.O.6′-neolignan | [59] |
| **109** | *rel*-(7*R*,8*S*,1'*R*,2'*R*,3'*S*,4'*S*,5'*R*)-7,2'-epoxy-2',3',4'-trihydoxy-1',2',3',4',5',6'-hexahydro-3,4,5'-trimethoxy-8,1'-neolign-8'-ene | *rel*-(7*R*,8*S*,1′*R*,3′*R*,4′*S*,5′*S*,6′*R*)-$\Delta^{8'}$-4′,5′,6′-trihydroxy-3,4,3′-trimethoxy-8.1′,7.O.6′-neolignan | [59] |
| **110** | *rel*-(7*R*,8*S*,1'*R*,2'*R*,3'*R*,4'*R*,5'*R*)-7.2',3'.4'-diepoxy-1',2',3',4',5',6'-hexahydro-2'-hydoxy-3,4,5'-trimethoxy-8,1'-neolign-8'-ene | *rel*- (7*R*,8*S*,1′*R*,3′*R*,4′R,5′R,6′R)-$\Delta^{8'}$-4′,5′-epoxy-6′-hydroxy-3,4,3′-trimethoxy-8.1′,7.O.6′-neolignan | [59] |
| **111** | *rel*-(7*R*,8*S*,1'*R*,2'*S*,5'*R*)-1',2',5',6'-tetrahydro-3,4,2',5'-tetramethoxy-8,1'-neolign-8'-ene | *rel*-(7*R*,8*S*,1′*R*,3′*R*,6′*S*)-$\Delta^{8'}$-3,4,3′,6′-tetramethoxy-8.1′, 7.O.6′~neolignan | [59] |
| **112** | *rel*-(7*R*,8*S*,1'*R*,5'*R*)-5',6'-dihydro-7-hydoxy-3,4,5'-trimethoxy-8,1'-neolign-8'-ene-2'(1'H)-one | *rel*-(7*R*,8*S*,1'*R*,5'*R*)-$\Delta^{8'}$-7-hydoxy-3,4,5'-trimethoxy-1′,2′,5',6'-tetrahydro-2′-oxo-8,1'-neolign | [57] |
| **113** | (8*S*,1'*R*,5'*R*)-5',6'-dihydro-3,4,5'-trimethoxy-8,1'-neolign-8'-ene-7,2'(1'H)-dione | (8*S*,1'*R*,5'*R*)-$\Delta^{3,8'}$- 3,4,5'-trimethoxy-7,2'-dioxo-8.1'-neolignan | [58] |

**Table SI-3.** Semi-systematic names and names in references of 8,3'-neolignans

| No. | Semi-systematic names | Names in references | Ref. |
|---|---|---|---|
| **130** | (7*S*,8*S*,1'*S*)-7,4'-epoxy-3,4,1'-trimethoxy-8,3'-neolign-8'-ene-6'(1'H)-one | (2*S*,3*S*,5*S*)-5-allyl-5-methoxy-3-methyl-2-veratryl-2,3,5,6-tetrahydro-6-oxobenzofuran | [40] |
| **131** | (7*S*,8*S*,1'*S*)-7,4'-epoxy-1'-methoxy-3,4-methylenedioxy-8,3'-neolign-8'-ene-6'(1'H)-one | (2*S*,3*S*,5*S*)-5-allyl-5-methoxy-3-methyl-2-(3′,4′-methylenedioxyphenyl)-2,3,5,6 -tetrahydro-6-oxobenzofuran | [62] |
| **135** | (7*S*,8*S*,1'*R*)-7,4'-epoxy-3,4,5,1'-tetramethoxy- 8,3'-neolign-8'-ene-6'(1'H)-one | (2*S*,3*S*,5*R*)-5-allyl-5-methoxy-3-methyl-2-(3′,4′,5′-trimethoxyphenyl)-2,3,5,6 -tetrahydro-6-oxobenzofuran | [64] |
| **136** | (7*S*,8*S*,1'*R*)-7,4'-epoxy-1'-methoxy-3,4-methylenedioxy-8,3'-neolign-8'-ene- 6'(1'H)-one | (7*S*,8*S*,1'*R*)-$\Delta^{8'}$-l′-methoxy-3,4-methylenedioxy-1′,6′-dihydro-6′-oxo-7.O.4′,8.3′-neolignan | [16] |
| **137** | (7*S*,8*S*,1'*R*)-7,4'-epoxy-1',5'-dimethoxy-3,4-methylenedioxy-8,3'-neolign-8'-ene- 6'(1'H)-one | (7*S*,8*S*,1'*R*)-$\Delta^{8'}$-l′,5′-dimethoxy-3,4-methylenedioxy-1′,6′-dihydro-6′-oxo-7.O.4′,8.3′-neolignan | [16] |
| **138** | (7*S*,8*S*,1'*R*)-7,4'- Epoxy-3,1'-dimethoxy-4,5-methylenedioxy-8,3'-neolign-8'-ene-6'(1'H)-one | (7*S*,8*S*,1'*R*)-$\Delta^{8'}$-3,l′-dimethoxy-3,4-methylenedioxy-1′,6′-dihydro-6′-oxo-7.O.4′,8.3′-neolignan | [25] |
| **139** | (7*S*,8*S*,1'*R*)-7,4'-epoxy-3,4,5,1',5'-pentamethoxy-8,3'-neolign-8'-ene-6'(1'H)-one | (2*S*,3*S*,5*R*)-5-allyl–5,7-dimethoxy-2-( 3′,4′,5′-trimethoxyphenyl)-3-methyl-2,3,5,6-tetrahydro-6-oxobenzofuran | [46] |
| **141** | (7*R*,8*S*,1'*R*)-7,4'-epoxy-3,5'-dimethoxy-4,5-methylenedioxy-8,3'-neolign-8'-ene-6'(1'H)-one | (2*R*,3*S*,5*R*)-5-allyl-5-methoxy-2-(3′-methoxy-4′,5′-methylenedioxyphenyl)-3-methyl-2,3,5,6-tetrahydro-6-oxobenzofuran | [46] |
| **143** | (7*S*,8*S*)-7,2'-epoxy-4'-hydroxy-3,5'-dimethoxy-4,5-methylenedioxy-8,3'-neolign-8'-ene | (2*S*,3*S*)-7-allyl-6-hydroxy-5-methoxy-2-(3-methoxy-4,5-methylenedioxyphenyl)-3-methyl-2,3 dihydrobenzofuran | [46] |
| **144** | (7*R*,8*S*)-7,2'-epoxy-6'-hydroxy-3,5'-dimethoxy-4,5-methylenedioxy-8,3'-neolign-8'-ene | (2*R*,3*S*)-7-allyl-6-hydroxy-5-methoxy-2-(3-methoxy-4,5-methylenedioxyphenyl)-3-methyl-2,3 dihydrobenzofuran | [46] |
| **145** | 7,2'-epoxy-6'-hydroxy-3,5'-dimethoxy-3,4-methylenedioxy-8,3'-neolign-7,8'-diene | 7-allyl-6-hydroxy-5-methoxy-2-(3′-methoxy-4′,5′-methylenedioxyphenyl)-3-methylbenzofuran | [46] |
| **146** | 7,2',-epoxy-6'-hydroxy-3,4,5,5'-tetramethoxy-8,3'-neolign-7,8'-diene | 7-allyl-6-hydroxy-5-methoxy-2-(3′,4′,5′-trimethoxyphenyl)-3-methylbenzofuran | [64] |
| **147** | 7,2'-epoxy-5'-hydroxy-3,4-dimethoxy-8,3'-neolign-7, 8'-diene | $\Delta^{7,8'}$-3′-hydroxy-3, 4-dimethoxy-8.1′,7.O.6′-7′:1′→5′ neolignan | [59] |
| **148** | 7,2'-Epoxy-6'-hydroxy-5'-methoxy-3,4-methylenedioxy-8,3'-neolign-7,8'-diene | 7-allyl-6-hydroxy-5-methoxy-3-methyl-2-piperonylbenzofuran | [42] |
| **155** | (8*S*)-6-hydroxy-5'-methoxy-3,4-methylenedioxy-8,3'-neolign-8'-ene-7-one | (8*S*)-$\Delta^{8'}$-4'-hydroxy-3'-methoxy-3,4-methylenedioxy-7-oxo-8.1',7':l'→5'-neolignan | [58] |
| **156** | *rel*-(8*R*,1'*R*,3'*R*)-2',3'-Dihydro- 3,4,5-trimethoxy-3',4'-methylenedioxy-8,3'-neolign-7-ene-6'(1H)-one | $\Delta^{8'}$-3,4,5-trimethoxy-3′,6′-dihydro-3′,4′-methylene-dioxy-6-oxo-8,3′-neolignan | [69] |
| **162** | *rel*-(7*S*,8*S*)-7,4'-epoxy-4-hydroxy-3,5'-dimethoxy-8',9'-bisnor-8,3'-neolign-7'-one | *rel*-(7S,8S)-4-hydroxy-3,3′-dimethoxy-7′-oxo-8,5′,7.O.4-8′,9′-bisnorneolignan | [59] |

**Table SI-4.** Semi-systematic names and names in references of cycloneolignans

| No. | Semi-systematic names | Names in references | Ref. |
|---|---|---|---|
| **174** | *rel*-(7*S*,8*R*,1'*R*,2'*R*,3'*S*)-1',2'-dihydro-2',5'-dihydroxy-3,3'-dimethoxy-4,5-methylenedioxy-7.3',8.1'-cycloneolign-8'-ene-4'(3'H)-one | *rel*-(1′*R*,5′*S*,6′R,7S,8R)-$\Delta^{2',8'}$-3′,6′-dihydroxy-3,5′-dimethoxy-4,5-methylenedioxy-4′-oxo-8.1′,7.5′-neolignan | [75] |
| **175** | *rel*-(7*S*,8*R*,1'*R*,2'*R*,3'*S*)-1',2'-dihydro-2'-hydroxy-3,3',5'-trimethoxy-4,5-methylenedioxy-7.3',8.1'-cycloneolign-8'-ene-4'(3'H)-one | *rel*-(1′*R*,5′*S*,6′R,7S,8R)-$\Delta^{2',8'}$-6′-hydroxy-3,3′,5′-trimethoxy-4,5-methylenedioxy-4′-oxo-8.1′,7.5′-neolignan | [75] |
| **176** | *rel*-(7*S*,8*R*,1'*R*,2'*R*,3'*S*)-2'-acetoxy-1',2'-dihydro-3,3',5'-tetramethoxy-4,5-methylenedioxy-7.3',8.1'-cycloneolign-8'-ene-4'(3'H)-one | *rel*-(1′*R*,5′*S*,6′R,7S,8R)-$\Delta^{2',8'}$-6′-acetoxy-3,3′,5′-trimethoxy-4,5-methylenedioxy-4′-oxo-8.1′,7.5′-neolignan | [64] |
| **177** | *rel*-(7*R*,8*R*,1'*R*,2'*R*,3'*R*)-2'-acetoxy-1',2'-dihydro-5'-methoxy-3,4-methylenedioxy-7.3',8.1'-cycloneolign-8'-ene-4'(3'H)-one | *rel*-(7*R*,8*R*,1′*R*,5′*R*,6′*R*)-$\Delta^{2',8'}$-6′-acetoxy-3′-methoxy-3,4-methylenedioxy-4′-oxo-8.1′-neolignan | [59] |
| **178** | *rel*-(7*S*,8*R*,1'*R*,3'*R*,4'*S*)-4'-acetoxy-3',4'-dihydro-3,4,5,3',5'-pentamethoxy-7.3',8.1'-cycloneolign-8'-ene-2'(1'H)-one | *rel*-(7*S*,8*R*,1′*R*,3′*R*,4′*S*)-$\Delta^{8'}$-4′-acetoxy-3,4,5,3′,5′ pentamethoxy-1′,2′,3′,4′-tetrahydro-5′-oxo-7 3′, 8 1′-neolignan | [76] |
| **179** | *rel*-(7*S*,8*R*,1'*R*,3'*R*,4'*S*)-4'-acetoxy-3',4'-dihydro-4-hydroxy-3,3',5'-trimethoxy-7.3',8.1'-cycloneolign-8'-ene-2'(1'H)-one | *rel*-(1′*R*,4′*S*,5′*R*,7*S*,8*R*)-$\Delta^{2',8'}$-4′-acetoxy-4-hydroxy-3,3′,5′-trimethoxy-6′-oxo-8.1′,7.5′-neolignan | [75] |
| **180** | *rel*-(7*S*,8*R*,1'*R*,3'*R*,4'*S*)-4'-acetoxy-3',4'-dihydro-3,3',5'-trimethoxy-4,5-methylenedioxy-7.3',8.1'-cycloneolign-8'- ene-2'(1'H)-one | *rel*-(1′*R*,4′*S*,5′*R*,7*S*,8*R*)-$\Delta^{2',8'}$-4′-acetoxy-3,3′,5′-trimethoxy-4,5-methylenedioxy-6′-oxo-8.1′,7.5′-neolignan | [75] |
| **181** | *rel*-(7*S*,8*R*,1'*R*,3'*R*,4'*S*)-4'-acetoxy-3',4'-dihydro-4-hydroxy-3,5,3',5'-tetra methoxy-7.3',8.1'-cycloneolign-8'-ene- 2'(1'H)-one | *rel*-(1′*R*,4′*S*,5′*R*,7*S*,8*R*)-$\Delta^{2',8'}$-4′-acetoxy-4-hydroxy-3,5,3′,5′-tetramethoxy-6′-oxo-8.1′,7.5′-neolignan | [75] |
| **182** | *rel*-(7*S*,8*R*,1'*R*,2'*R*,3'*S*,4'*S*)-4'-acetoxy-1',2',3', 4'-tetrahydro-2'-hydroxy-3',5'-dimethoxy-3,4-methylenedioxy-7.3',8.1'-cycloneolign-8'-ene | *rel*-(7*S*,8*R*,1′*R*,2′*R*,3′*S*,4′*S*)-$\Delta^{8'}$-4′-acetoxy-2′-hydroxy-3′,5′-dimethoxy-3,4-methylenedioxy-1′,2′,3′,4 -tetrahydro-7 3′,8 1′-neolignan | [76] |
| **183** | *rel*-(7*S*,8*R*,1'*R*,2'*R*,3'*S*,4'*S*)-4'-acetoxy-1',2',3',4'-tetrahydro-2'-hydroxy-3,3',5'-trimethoxy-4,5-methylenedioxy-7.3',8.1'-cycloneolign-8'-ene | *rel*-(7*S*,8*R*,1′*R*,2′*R*,3′*S*,4′*S*)-$\Delta^{8'}$-4′-acetoxy-2′-hydroxy-3,3′,5′-trimethoxy-3,4-methylenedioxy-1′,2′,3′,4′-tetrahydro-7 3′,8 1′-neolignan | [76] |
| **184** | *rel*-(7*S*,8*R*,1'*S*,2'*R*,3'*S*,4'R)-4'-acetoxy-1',2',3',4'-tetrahydro-2'-hydroxy-3'-methoxy-3,4-methylenedioxy-7.3',8.1'-cycloneolign-8'-ene-5'(6'H)-one | *rel*-(7*S*,8*R*,1'*S*,2'*R*,3'*S*,4'R)-$\Delta^{8'}$-4'-acetoxy-2′-hydroxy-3′-methoxy-3,4-methylenedioxy-1′,2′,3′,4′,5′,6′-hexahydro-5′-oxo-7 3′, 8 1′-neolignan | [46] |
| **185** | *rel*-(7*S*,8*R*,1'*R*,2'*R*,3'*S*,4'R)-4'-acetoxy-1',2',3',4'-tetrahydro-2'-hydroxy-3',3-dimethoxy-4,5-methylenedioxy-7.3',8.1'-cycloneolign-8'-ene-5'(6'H)-one | *rel*-(7*S*,8*R*,1'*S*,2'*R*,3'*S*,4'R)-$\Delta^{8'}$-4'-acetoxy-2′-hydroxy-3, 3′-dimethoxy-3,4-methylenedioxy-1′,2′,3′,4′,5′,6′-hexahydro-5′-oxo-7 3′, 8 1′-neolignan | [46] |
| **186** | *rel*-(7*S*,8*R*,1'*S*,3'*S*,4'*S*)-4'-acetoxy-3',4'-dihydro-4-hydroxy-3,3',5'-trimethoxy-7.3',8.1'-cycloneolign-8'-ene-2'(1'H)-one | *rel*-(1′*R*,4′*R*,5′*R*,7*R*,8*S*)-$\Delta^{2',8'}$-4′-acetoxy-4-hydroxy-3,3′,5′-trimethoxy-6′-oxo-8.1′,7.5′-neolignan | [75] |
| **187** | *rel*-(7*S*,8*R*,1'*S*,3'*S*,4'*S*)-4'-acetoxy-3',4'-dihydro-3,4,3',5'-tetramethoxy-7.3',8.1'-cycloneolign-8'-ene-2'(1'H)-one | *rel*-(1′*R*,4′*R*,5′*R*,7*R*,8*S*)-$\Delta^{2',8'}$-4′-acetoxy-3,4,3′,5′-tetramethoxy-6′-oxo-8.1′,7.5′-neolignan | [75] |

| | | | |
|---|---|---|---|
| **188** | (7*S*,8*R*,1'*S*,3'*S*,4'*S*)-3',4'-dihydro-4,4'-dihydroxy-3,3',5'-trimethoxy- 7.3',8.1'-cycloneolign-8'-ene-2'(1'H)-one | (7*S*,8*R*,1′*S*,3′*S*,4′*S*)-$\Delta^{8'}$-4,4′-dihydroxy-3,3′,5′-trimethoxy-1′,2′,3′,4′-tetrahydro-2′-oxo-7.3′,8.1′-neolignan | [16] |
| **189** | *rel*-(7*S*,8*R*,1'*S*,3'*S*,4'*S*)-3',4'-dihydro-4'-hydroxy-3,3',5'-trimethoxy-4,5-methylenedioxy-7.3',8.1'-cycloneolign-8'-ene-2'(1'H)-one | *rel*-(7*S*,8*R*,1′*S*,4′*R*,*S*′*R*)-4′-hydroxy-3,3′,5′-trimethoxy-4,5-methylenedioxy-6′-oxo-Δ-1,3,5,2′,8′-8.1′,7.5′-neolignan | [78] |
| **190** | *rel*-(7*R*,8*R*,1'*S*,3'*R*,4'*R*)-3',4'-dihydro-4'-hydroxy-5'-methoxy-3,4-methylenedioxy-7.3',8.1'-cycloneolign-8'-ene-2'(1'H)-one | *rel*-(7*S*,8*S*,1′*R*,4′*S*,5′*S*)-$\Delta^{2',8'}$-4′-hydroxy-3′-methoxy-3,4-methylenedioxy-6′-oxo-8.1′,7.5′-neolignan | [59] |
| **191** | *rel*-(7*R*,8*R*,1'*R*,3'*R*)-4-hydroxy-3,5'-dimethoxy-7.3',8.1'-cycloneolign-8'-ene-2',4'(1'H,3'H)-dione | (1*R*,5*R*,6*R*,7*R*)-1-allyl-6-(4′-hydroxy-3′-methoxy phenyl)-3-methoxy-7-methyl-4,8-dioxobicyclo[3,2,1] oct-2-ene | [62] |
| **192** | (7*R*,8*R*,1'*S*,2'*R*,3'*S*)-2'-hydroxy-5'-methoxy-3,4-methylenedioxy-1',2'-dihydro-7.3',8.1'-cycloneolign-8'-ene-4(3'H)-one | (7*R*,8*R*,1'*S*,2'*R*,3'*S*)-$\Delta^{5',8'}$-2'-hydroxy-6'-methoxy-3,4-methylenedioxy-4'-oxo-7.3',8.1'-neolignan | [58] |
| **193** | *rel*-(7*S*,8*R*,1'*S*,2'*S*,3'*R*)-2'-acetoxy-1',2'-dihydro-5'-hydroxy-3'-methoxy-3,4-methylenedioxy-7.3',8.1'-cycloneolign-8'-ene-4'(3'H)-one | *rel*-(1*S*,5*S*,6*S*,7*R*,8*R*)-8-acetoxy-1-allyl-3-hydroxy-5-methoxy-7-methyl-4-oxo-6-piperonylbicyclo[3.2,1] oct-2-ene | [40] |
| **194** | *rel*-(7*S*,8*R*,1'*S*,2'*S*,3'*R*)-2'-acetoxy-1',2'-dihydro-3',5'-dimethoxy-3,4-methylenedioxy- 7.3',8.1'-cycloneolign-8'-ene-4'(3'H)-one | *rel*-(1*S*,5*S*,6*S*,7*R*,8*R*)-8-acetoxy-1-allyl-3, 5-dimethoxy-7-methyl-4-oxo-6-piperonylbicyclo[3.2,1]oct-2-ene | [40] |
| **195** | *rel*-(7*S*,8*R*,1'*S*,2'*S*,3'*R*)-1',2'-dihydro-2'-hydroxy-3',5'-dimethoxy-3,4-methylenedioxy-7.3',8.1'-cycloneolign-8'-ene-4'(3'H)-one | *rel*-(1*S*,5*S*,6*S*,7*R*,8*R*)-1-allyl-8-hydroxy-3,5-dimethoxy-7-methyl-4-oxo-6-piperonylbicyclo[3,2,1]oct-2-ene | [40] |
| **196** | *rel*-(7*S*,8*R*,1'*S*,2'*S*,3'*R*)-2'-acetoxy-1',2'-dihydro-3,4,3',5'-tetramethoxy-7.3',8.1'-cycloneolign-8'-ene-4'(3'H)-one | (7*S*,8*R*,1'*S*,2'*S*,3'*R*)-$\Delta^{8'}$-2'-acetoxy-3',5',3,4-tetramethoxy-1',2',3',4'-tetrahydro-4'-oxo-7.3',8.1'-neolignan | [65] |
| **197** | *rel*-(7*S*,8*R*,1'*S*,2'*S*,3'*R*)-2'-acetoxy-1',2'-dihydro-3,4,5,3',5'-pentamethoxy-7.3',8.1'-cycloneolign-8'-ene -4'(3'H)-one | (7*S*,8*R*,1'*S*,2'*S*,3'*R*)-$\Delta^{8'}$-2'-acetoxy-3',5',3,4,5-pentamethoxy-1',2',3',4'-tetrahydro-4'-oxo-7.3',8.1'-neolignan | [65] |
| **198** | *rel*-(7*S*,8*R*,1'*S*,2'*S*,3'*R*)-2'-acetoxy-1',2'-dihydro-3,3',5'-trimethoxy-4,5-methylenedioxy-7.3',8.1'-cycloneolign-8'-ene-4'(3'H)-one | (7*S*,8*R*,1'*S*,2'*S*,3'*R*)-$\Delta^{8'}$-2'-acetoxy-3',5',3-trimethoxy-4,5-methylenedioxy-1',2',3',4'-tetrahydro-4'-oxo-7.3',8.1'-neolignan | [65] |
| **200** | (7*S*,8*R*,1'*S*,2'*S*,3'*R*)-1',2'-dihydro-2',5'-dihydroxy-3'-methoxy-3,4- methylenedioxy-7.3',8.1'-cycloneolign-8'-ene-4'(3'H)-one | (7*S*,8*R*,1′*S*,5*R*,6′*S*)-$\Delta^{2',8'}$-3',6'-dihydroxy-5'-methoxy-3,4-methylenedioxy-4′-oxo-8.1′,7.5′-neolignan | [45] |
| **201** | (7*S*,8*R*,1'*S*,2'*S*,3'*R*)-2'-acetoxy-1',2'-dihydro-3,3',5'-trimethoxy-4,5-methylenedioxy-7.3',8.1'-cycloneolign-8'-ene-4'(3'H)-one | (7*S*,8*R*,1′*R*,2′*R*,3′*S*)-$\Delta^{8'}$-2'-acetoxy-3,3',5'-trimethoxy-4,5-methylenedioxy-1',2',3',4'-tetrahydro-4'-oxo-7.3',8.1'-neolignan | [25] |
| **202** | *rel*-(7*S*,8*R*,1'*S*,2'*R*,3'*S*)-2'-acetoxy-1',2',5',6'-tetrahydro-3',5'-dimethoxy-3,4-methylenedioxy-7.3',8.1'-cycloneolign-8'-ene-4'(3'H)-one | (7*S*,8*R*,1'*R*,2'*R*,3'*S*)-$\Delta^{8'}$-2'-acetoxy-3',5'-dimethoxy-3,4-methylenedioxy-1',2',3',4'-tetrahydro-4'-oxo-7.3',8.1'-neolignan | [65] |
| **203** | *rel*-(7*S*,8*R*,1'*S*,2'*R*,3'*S*)-1',2',5',6'-tetrahydro-2'-hydroxy-3',5'-dimethoxy-3,4-methylenedioxy-7.3',8.1'-cycloneolign-8'-ene-4'(3'H)-one | (7*S*,8*R*,1'*R*,2'*R*,3'*S*)-$\Delta^{8'}$-3',5'-dimethoxy-2'-hydroxy-3,4-methylenedioxy-1',2',3',4'-tetrahydro-4'-oxo-7.3',8.1'-neolignan | [65] |

| | | | |
|---|---|---|---|
| **204** | *rel*-(7S,8R,1'S,2'R,3'S)-2'-acetoxy-1',2',5',6'-tetrahydro-3,4,3',5'-tetramethoxy-7.3',8.1'-cycloneolign-8'-ene-4'(3'H)-one | (7S,8R,1'R,2'R,3'S)-$\Delta^{8'}$-2'-acetoxy-3',5',3,4-tetramethoxy-1',2',3',4'-tetrahydro-4'-oxo-7.3',8.1'-neolignan | [65] |
| **205** | *rel*-(7S,8R,1'S,2'R,3'S)-1',2',5',6'-tetrahydro-4,2'-dihydroxy-3,3',5'-trimethoxy-7.3',8.1'-cycloneolign-8'-ene-4'(3'H)-one | (7S,8R,1'R,2'R,3'S)-$\Delta^{8'}$-2',4-dihydroxy-3,3',5'-trimethoxy-1',2',3',4'-tetrahydro-4'-oxo-7.3',8.1'-neolignan | [65] |
| **206** | *rel*-(7S,8R,1'S,2'S,3'R,4'R)-2'-acetoxy-1',2',3',4'-tetrahydro-4'-hydroxy-3',5'-drimethoxy-3,4-methylenedioxy-7.3',8.1'-cycloneolign-8'-ene | (7S,8R,1'S,2'S,3'R,4'S)-$\Delta^{8'}$-2'-acetoxy-3',5'-dimethoxy-4'-hydroxy-3,4-methylenedioxy-1',2',3',4'-tetrahydro-7.3',8.1'-neolignan | [65] |
| **207** | *rel*-(7S,8R,1'S,2'S,3'R,4'R)-2'-acetoxy-1',2',3',4'-tetrahydro-4'-hydroxy-3,4,3',5'-tetramethoxy-7.3',8.1'-cycloneolign-8'-ene | (7S,8R,1'S,2'S,3'R,4'S)-$\Delta^{8'}$-2'-acetoxy-3,4,3',5'-tetramethoxy-4'-hydroxy-1',2',3',4'-tetrahydro-7.3',8.1'-neolignan | [65] |
| **208** | (7S,8R,1'S,2'S,3'R,4'R)-1',2',3',4'- tetrahydro-2',4'-dihydroxy-3',5'-dimethoxy-3,4-methylenedioxy-7.3', 8.1'-cycloneolign-8'-ene | (7S,8R,l'S,2'S,3'R,4'R)-$\Delta^{8'}$-2',4'-dihydroxy-3',5'-dimethoxy-3,4-methylenedioxy-l',2',3',4'-tetrahydro-7.3',8.l'-neolignan | [16] |
| **209** | (7S,8R,1'S,2'S,3'R,4'S)-1',2',3',4'-tetrahydro-2',4'-dihydroxy-3',5'-dimethoxy-3,4-methylenedioxy-7.3', 8.1'-cycloneolign-8'-ene | (7S,8R,l'S,2'S,3'R,4'S)-$\Delta^{8'}$-2',4'-dihydroxy-3',5'-dimethoxy-3,4-methylenedioxy-l',2',3',4'-tetrahydro-7.3',8.l'-neolignan | [16] |
| **210** | (7S,8R,1'S,2'S,3'R,4'S)-1',2',3',4'-tetrahydro-2',4'-dihydroxy-5,3',5'-trimethoxy-3,4-methylenedioxy-7.3',8.1'-cycloneolign-8'-ene | (7S,8R,l'S,2'S,3'R,4'S)-$\Delta^{8'}$-2',4'-dihydro-3,3',5'-trimethoxy-4,5-methylenedioxy-l',2',3',4'-tetrahydr-7.3',8.l'-neolignin | [16] |
| **211** | *rel*-(7R,8R,1'S,2'R,3'S,4'R)-1',2',3',4'-tetrahydro-2',4'-dihydroxy-5'-methoxy-3,4-methylenedioxy-7.3',8.1'-cycloneolign-8'-ene | *rel*-(7S,8S,l'R,4'S,5'R,6'S)-$\Delta^{2',8'}$-4',6'-dihydroxp-3'-methoxy-3,4-methylenedioxy-8.1',7.5'-neolignan | [59] |
| **214** | (7R,8R,l'R,2'R,3'S,4'S)-1',2',3',4'-tetrahydro-2',4'-dihydroxy-3,4-methylenedioxy-7.3',8.1'-cycloneolign-8'-ene-5'(6'H)-one | (7R,8R,l'R,2'R,3'S,4'S)-$\Delta^{8'}$-2',4'-dihydroxy-3,4-methylenedioxy-l',2',3',4',5',6'-hexahydro-5'-oxo-7.3', 8.1'-neolignan | [43] |
| **215** | (7R,8R,l'R,2'R,3'S,4'S)-1',2',3',4'-tetrahydro-2'-hydroxy-4'-methoxy-3,4-methylenedioxy-7.3',8.1'-cycloneolign-8'-ene-5'(6'H)-one | (7R,8R,l'R,2'R,3'S,4'S)-$\Delta^{8'}$-2'-hydroxy-4'-methoxy -3,4-methylenedioxy-l',2',3',4',5',6'-hexahydro-5'-oxoneolignan | [43] |
| **216** | (7R,8R,l'R,2'R,3'S,4'R)-2'-hydroxy-3,4'-dimethoxy-4,5-methylenedioxy-1',2',3',4'-tetrahydro-7.3',8.1'-cycloneolign-8'-ene-5'(6'H)-one | (7R,8R,l'R,2'R,3'S,4'R)-$\Delta^{8'}$-2'-hydroxy-3,4'-dimethoxy-4,5-methylenedioxy-l',2',3',4',5',6'-hexahydro-5'-oxoneolignan | [43] |
| **217** | (7S,8R,l'R,2'S,3'R,4'R)-1',2',3',4'-tetrahydro-2',4'-dihydroxy-3,3'-dimethoxy-4,5-methylenedioxy-7.3',8.1'-cycloneolign-8'-ene-5'(6'H)-one | (7S,8R,l'R,2'S,3'R,4'R)-$\Delta^{8'}$-2',4'-dihydroxy-3,3'-dimethoxy-4,5-methylenedioxy-l',2',3',4',5',6' -hexahydro-5'-oxo-7.3',8.1'-neolignan | [77] |
| **218** | (7R,8R,1'R,3'S)-5',6'-dihydro-4-hydroxy-3,5'-dimethoxy-7.3',8.1'- cycloneolign-8'-ene-2',4'(1'H,3'H)-dione | (7R,8R,1'R,3'S)-$\Delta^{8'}$-4-hydroxy-3,5'-dimethoxy-l',2',3',4',5',6'-hexahydro-4'-oxo-7.3', 8.1'-neolignan | [43] |
| **219** | (7R,8S,l'R,2'S,3'S)-1',2'-dihydro-2'-hydroxy-3,3',5'-trimethoxy-4,5-methylenedioxy-7.3',8.1'-cycloneolign-8'-ene-4'(3'H)-one | (7R,8S,l'R,2'S,3'S)-$\Delta^{8'}$-2'-hydroxy-3,3',5'-trimethoxy-4,5-methylenedioxy-l',2',3',4'-tetrahydro-4'-oxo-7.3', 8.1'-neolignan | [50] |
| **220** | *rel*-(7S,8S,l'R,2'S,3'R)-2'-acetoxy-1',2'-dihydro-5'-methoxy-3,4-methylenedioxy-7.3',8.1'-cycloneolign-8'-ene-4'(3'H)-one | *rel*-(7S,8S,1'R,5'R,6'S)-$\Delta^{2',8'}$-6'-acetoxy-3'-methoxy-3,4-methylenedioxy-4'-oxo-8.1',7.5'-neolignan | [59] |

| | | | |
|---|---|---|---|
| **221** | *rel*-(7*S*,8*R*,1'*R*,3'*S*,4'*S*,5'*S*)-3',4',5',6'-tetrahydro-4'-hydroxy-3,3',5'-trimethoxy-4,5-methylenedioxy-7.3', 8.1' -cycloneolign-8'-ene-2'(1'H)-one | *rel*-(7*S*,8*R*,1'*R*,3'*S*,4'*S*,5'*S*)-$\Delta^{8'}$-4′-hydroxy-3,3′,5′-trimethoxy-4,5-methylenedioxy-1′,2′,3′,4′,5′,6′-hexahydro-2′-oxo-7.3′,8.1′-neolignan | [77] |
| **222** | *rel*-(7*S*,8*R*,1'*R*,3'*S*,4'*S*,5'*S*)-3',4',5',6'-tetrahydro-4'-hydroxy-3,4,5, 3',5'-pentamethoxy-7.3',8.1'-cycloneolign-8'-ene-2'(1'H)-one | *rel*-(7*S*,8*R*,1'*R*,3'*S*,4'*S*,5'*S*)-$\Delta^{8'}$-4′-hydroxy-3,4,5,3′,5′-pentamethoxy-1′,2′,3′,4′,5′,6′-hexahydro-2′-oxo-7.3′,8.1′-neolignan | [77] |
| **223** | *rel*-(7*R*,8*R*,1'*R*,3'*R*,4'*R*, 5'*S*)-3',4',5',6'-tetrahydro-4'-hydroxy-5'-methoxy-3,4-methylenedioxy-7.3',8.1'-cycloneolign-8'-ene- 2'(1'H)-one | *rel*-(7*S*,8*S*,1′*S*,3′*R*,4′*S*,5′*S*)-$\Delta^{8'}$-4′-hydroxy-3′-methoxy-3,4-methylenedioxy-6′-oxo-8.1′,7.5′-neolignan | [59] |
| **224** | (7*R*,8*S*,1'*S*,2'*S*,3'*S*,4'*R*)-1',2',3',4'-tetrahydro-2',4'-dihydroxy-3,3'-dimethoxy-4,5-methylenedioxy-7.3',8.1'-cycloneolign-8'-ene-5'(6'H)-one | (7*R*,8*S*,1'*S*,2'*S*,3'*S*,4'*R*)-$\Delta^{8'}$-2',4'-dihydroxy-3,3'-dimethoxy-4,5-methylenedioxy-1′,2′,3′,4′,5′,6′-hexahydro-5′-oxo-7.3′,8.1′-neolignan | [50] |
| **227** | *rel*-(7*R*,8*R*,1'*R*,2'*R*,3'*S*,4'*R*,5'*R*)-1',2',3',4',5',6'-hexahydro-2', 4'-diydroxy-5'-methoxy-3,4-methylenedioxy-7.3',8.1'-cycloneolign-8'-ene | *rel*-(7*S*,8*S*,1′*S*,3′*R*,4′*S*,5′*S*,6′*S*)-$\Delta^{8'}$-4′,6′-dihydroxv-3′-methoxy-3,4-methylenedioxy-8.1′,7.5′-neolignan | [59] |
| **228** | (7*R*,8*R*,1'*S*,3'*S*)-4-hydroxy-3,5'-dimethoxy-7.3',8.1'-cycloneolign-8'-ene-2',4'(1'H,3'H)-dione | (7*R*,8*R*,1'*S*,3'*S*)-$\Delta^{8'}$-4-hydroxy-3,5'-dimethoxy-1',2',3', 4'-tetrahydro-4'-oxo-7.3',8.1'-neolignan | [43] |
| **229** | *rel*-(7*R*,8*R*,1'*S*,3'*S*)-5'-methoxy-3,4-methylenedioxy -7.3',8.1'-cycloneolign-8'-ene-2',4'(1'H,3'H)-dione | *rel*-(1*R*,5*S*,6*R*,7*R*)-1-allyl-3-methoxy-7-methyl-4,8-dioxo-6-piperonylbicyclo[3,2,l]oct-2-ene | [69] |
| **230** | (7*R*,8*R*,1'*S*,3'*S*)-3,5'-dimethoxy-4,5-methylenedioxy-7 .3',8.1'-cycloneolign-8'-ene-2',4'(1'H,3'H)-dione | (7*S*,8*R*,1′*R*,3′*S*)-$\Delta^{8'}$-3,5′-dimethoxy-4,5-methylenediox y-1′,2′,3′,4′-tetrahydro-2′,4′-dioxo-7,3′.8,1′-neolignan | [25] |
| **231** | (7*R*,8*R*,1'*S*,3'*S*)-3,4,5,5'-tetramethoxy-3,4-methylenedioxy-7.3',8.1'-cycloneolign-8'-ene-2',4' (1'H, 3'H)-dione | (7*S*,8*R*,1′*R*,3′*S*)-$\Delta^{8'}$-3,4,5,5′-tetramethoxy-1′,2′,3′,4′-tetr ahydro-2′,4′-dioxo-7.3′,8.1′-neolignan | [25] |
| **232** | (7*S*,8*R*,1'*R*,2'*S*,3'*R*)-2'-acetoxy-1',2',5',6'-tetrahydro-3',5'-dimethoxy-3,4-methylenedioxy-7.3,8.1'-cycloneolign-8'-ene-4'(3'H)-one | (7*S*,8*R*,1'*S*,2'*S*,3'*R*)-$\Delta^{8'}$-1′,2′,3′,4′-tetrahydro-4'-oxo-7.3' ,8.1'-neolignan | [80] |
| **246** | (7*R*,8*R*,3'*S*,5'*S*)-3,4,5,5′-tetramethoxy-7.3',8.5'-cyclone olign-8'-ene-2',4'(3'H,5'H)-dione | (7*R*,8*R*,3'*S*,5'*S*)-$\Delta^{8'}$-3,4,5,5′-tetramethoxy-2′,3′,4′,5′-tetr ahydro-2′,4′-dioxo-7.3′,8.5′-neolignan | [28] |

**Table 5.** Semi-systematic names and names in references of uncommon lignans

| No. | Semi-systematic names | Names in references | Ref. |
|---|---|---|---|
| **258** | (7*R*,8*S*)-7,5'-Epoxy-3,2'-dimethoxy-4,5-methylenedioxy-1',7'-seco-8,4'-neolign-8'-ene-1',7'-ether | (2*R*,3*S*)-6-O-allyl-5-methoxy-2-(3′-methoxy-4′,5′-methylenedioxyphenyl)-3-methyl-2,3-dihydrobenzofuran | [46] |
| **259** | (7*S*,8*S*)-7,5'-epoxy-3,2'-dimethoxy-4,5-methylenedioxy-1',7'-seco-8,4'-neolign-8'-ene-1',7'-ether | (2*S*,3*S*)-6-O-a11y1-5-methoxy-2-(3′-methoxy-4′,5′-methylenedioxyphenyl)-3-methyl-2,3-dihydrobenzofuran | [46] |
| **260** | (7*S*,8*S*)-7,5'-epoxy-3,4,5,2'-tetramethoxy-1',7'-seco-8,4'-neolign-8'-ene-1',7'-ether | (2*S*,3*S*)-6-O-allyl-5-methoxy-2-(3′,4′,5′-trimethoxyphenyl)-3-methyl-2,3-dihydrobenzofuran | [64] |
| **261** | (7*S*,8*S*)-7,5'-epoxy-3,4,5,2',5'-pentamethoxy-1',7'-seco-8,4'-neolign-8'-ene-1',7'-ether | (2*S*,3*S*)-6-O-allyl-5,7-dimethoxy-2-(3′,4′,5′-trimethoxyphenyl)-3-methyl-2,3-dihydrobenzofuran | [46] |
| **262** | (7*S*,8*S*)-7,5'-epoxy-2'-methoxy-4,5-methylenedioxy-1',7'-seco-8,4'-neolign-8'-ene-1',7'-ether | (2*S*,3*S*)-6-O-allyl-5-methoxy-3-methyl-2-piperonyl-2,3-dihydrobenzofuran | [42] |
| **263** | (7*S*,8*S*)-7,5'-epoxy-2',6'-dimethoxy-4,5-methylenedioxy-1',7'-seco-8,4'-neolign-8'-ene-1',7'-ether | (7*S*,8*S*)-$\Delta^{1',3',5',8'}$-3′,5′-trimethoxy-3,4-methylenedioxy-8.1′,7.O.6′,4′.O.7′-neolignan | [45] |
| **264** | (7*S*,8*S*)-7,5'-epoxy-3,2',6'-trimethoxy-4,5-methylenedioxy-1',7'-seco-8,4'-neolign-8'-ene-1',7'-ether | (7*S*,8*S*)-$\Delta^{1',3',5',8'}$-5,3′,5′-trimethoxy-3,4-methylenedioxy-8.1′,7.O.6′,4′.O.7′-neolignan | [45] |
| **267** | *rel*-2'-methoxy-3,4-methylenedioxy-1',7'-seco-8,4'-neolign-8'-ene-1',7'- ether | *rel*-(8S)-$\Delta^{8'}$-3′-methoxy-3,4-methylenedioxy-7-oxo-8.1′,7′:1′O.4′-neolignan | [59] |
